# Sulfur transfer and activation by ubiquitin-like modifier system Uba4•Urm1 link protein urmylation and tRNA thiolation in yeast

**DOI:** 10.15698/mic2016.11.539

**Published:** 2016-10-24

**Authors:** André Jüdes, Alexander Bruch, Roland Klassen, Mark Helm, Raffael Schaffrath

**Affiliations:** 1 Universität Kassel, Institut für Biologie, FG Mikrobiologie, Heinrich-Plett-Str. 40, 34132 Kassel, Germany.; 2 Johannes Gutenberg Universität Mainz, Institut für Pharmazie und Biochemie, Staudinger Weg 5, 55128 Mainz, Germany.

**Keywords:** Saccharomyces cerevisiae, ubiquitin-like modifier Urm1, protein urmylation, tRNA thiolation, E1-like enzyme Uba4, sulfur transferase Tum1, tRNase zymocin

## Abstract

Urm1 is a unique dual-function member of the ubiquitin protein family and conserved from yeast to man. It acts both as a protein modifier in ubiquitin-like urmylation and as a sulfur donor for tRNA thiolation, which in concert with the Elongator pathway forms 5-methoxy-carbonyl-methyl-2-thio (mcm^5^s^2^) modified wobble uridines (U34) in anticodons. Using *Saccharomyces cerevisiae* as a model to study a relationship between these two functions, we examined whether cultivation temperature and sulfur supply previously implicated in the tRNA thiolation branch of the *URM1 *pathway also contribute to proper urmylation. Monitoring Urm1 conjugation, we found urmylation of the peroxiredoxin Ahp1 is suppressed either at elevated cultivation temperatures or under sulfur starvation. In line with this, mutants with sulfur transfer defects that are linked to enzymes (Tum1, Uba4) required for Urm1 activation by thiocarboxylation (Urm1-COSH) were found to maintain drastically reduced levels of Ahp1 urmylation and mcm^5^s^2^U34 modification. Moreover, as revealed by site specific mutagenesis, the S-transfer rhodanese domain (RHD) in the E1-like activator (Uba4) crucial for Urm1-COSH formation is critical but not essential for protein urmylation and tRNA thiolation. In sum, sulfur supply, transfer and activation chemically link protein urmylation and tRNA thiolation. These are features that distinguish the ubiquitin-like modifier system Uba4•Urm1 from canonical ubiquitin family members and will help elucidate whether, in addition to their mechanistic links, the protein and tRNA modification branches of the *URM1* pathway may also relate in function to one another.

## INTRODUCTION

In eukaryotes, the activity of many diverse proteins can be modulated by conjugation to ubiquitin and ubiquitin-like modifiers 1,2. Among the latter, Urm1 from *Saccharomyces cerevisiae* 3 is unique since it can act as a lysine-directed protein modifier in ubiquitin-like urmylation and as a sulfur donor for tRNA anticodon thiolation 4,5,6,7,8,9. Both roles are exchangeable among eukaryotes 10,11 and based on dual-function Urm1-like proteins in archaea and bacteria 12,13, conserved Urm1 systems appear to be important in all domains of life. In line with this, their inactivation triggers stress-induced growth defects in microbes, organ underdevelopment in plants and, strikingly, lethality in flies 3,4,8,12,13,14,15,16,17.

**Figure 1:**
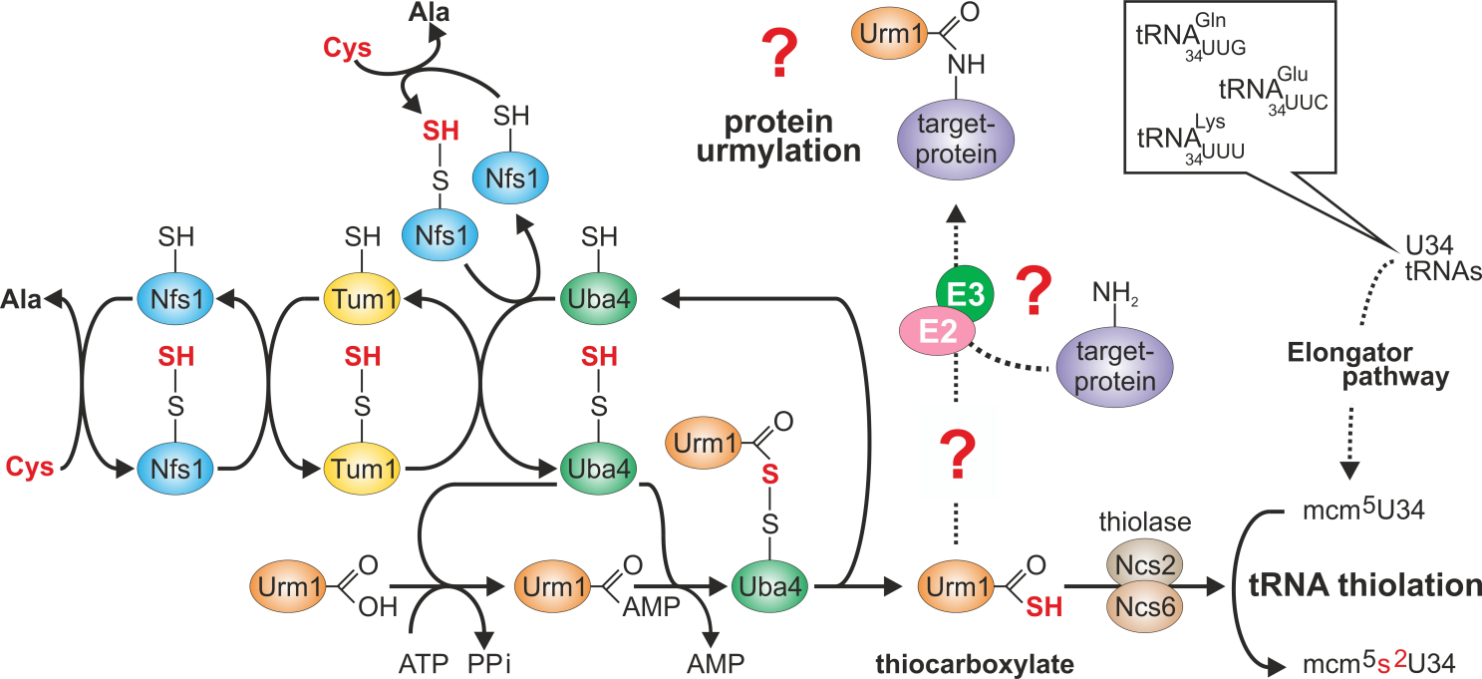
FIGURE 1: Sulfur flow within the *URM1 *pathway. The scheme depicts sulfur (red) flow and *URM1 *pathway players required for mobilization (Nfs1), transfer (Tum1), activation (Uba4, Urm1) or consumption (Ncs2•Ncs6) of sulfur. E1-like activator Uba4 (green) is key to Urm1 thiocarboxylation. Urm1-COSH formed this way donates sulfur to the tRNA thiolation branch which cooperates with the Elongator pathway to form 5-methoxy-carbonyl-methyl-2-thio-uridine in wobble positions (mcm^5^s^2^U34) of the indicated tRNA anticodons. Possibly (?) Urm1-COSH also feeds into the urmylation branch of the* URM1* pathway. As for the latter, E2/E3 enzymes are elusive (?) and the relevance of urmylation for target protein function is ill-defined (?). The model is up-dated from work in the labs of Hayashi 7 and Suzuki 9.

In contrast to ATP-dependent ubiquitin adenylation and conjugation by E1-E3 enzymes, E2/E3 activities for Urm1 are unknown, and Urm1 activation by its E1-like enzyme Uba4 results in C-terminal thiocarboxylation (Urm1-COSH) 18,19. This is similar to E1-like (MoeB or ThiF) activation of bacterial S-carrier proteins (MoaD or ThiS) that donate sulfur for molybdopterin or thiamine synthesis rather than being involved in protein conjugation 20,21. So in addition to eukaryal ubiquitin-like proteins, Urm1 relates to prokaryal S-carriers 19. In line with this, a conserved S-relay system contributes to Urm1-COSH formation (Fig. 1). It starts with desulfurase Nfs1 that mobilizes sulfur from cysteine for direct S-transfer onto Uba4 (Fig. 1) or indirectly via sulfur transferase Tum1 7,8,9,10. Uba4 is composed of MoeB-like (see above) and rhodanese-type domains (MoeBD, RHD) that carry thiol-active cysteines 3,8,18,22. S-transfer to the one in RHD results in a persulfide (Fig. 1) which following adenylation of Urm1 by the MoeBD forms an acyl-disulfide with the modifier 18,21. Upon reductive cleavage of this bond, Urm1-COSH gets released 18,23 to donate the activated sulfur species for S-insertion into tRNAs (Fig. 1) by thiolase (Ncs2•Ncs6) 7,8,9.

In concert with the conserved Elongator pathway, this generates 5-methoxy-carbonyl-methyl-2-thio (mcm^5^s^2^) modifications of wobble uridines (U34) in tRNA anticodons 24,25,26. These support codon-anticodon interaction for efficient mRNA decoding 27, which explains why mcm^5^s^2^U34 modification defects in Elongator and *URM1* pathway mutants cause translational defects such as frameshift errors, tRNA anti-suppression and ribosomal stalling 28,29,30. Evidence demonstrating that tRNA modifications including mcm^5^s^2^U34 can change in response to environmental cues suggests dynamics in their formation 31,32. Whether this is due to active regulation is an attractive option that is supported by recent data showing that Elongator and thiolase proteins undergo posttranslational modifications including reversible phosphorylation and intriguingly, urmylation itself 6,14,33,34,35,36.

As for its urmylation role, data obtained under steady-state-conditions suggest the major pool of non-conjugated Urm1 is in its thiocarboxylate form 6,37. So Urm1-COSH formation by Uba4 (Fig. 1) per se seems not to be sufficient for conjugation. However, when exposed to the thiol oxidizer diamide, Urm1-COSH generated *in vitro* becomes engageable in urmylation 6. Together with evidence that reactive oxygen species (ROS) and diamide induce urmylation in yeast and human cells, the S-carrier and protein modifier functions of Urm1 were proposed to be coupled to each other linking both to oxidative stress 4,6,11,37. In support of this is the evidence showing that ROS detoxifying peroxiredoxins are urmylated in yeast (Ahp1) and fruit flies (Prx5) 4,6,11,17.

Because of its dual-functionality Urm1 was coined a ubiquitin-like fossil at the crossroad of S-transfer and protein conjugation 8, thus deviating from canonical ubiquitination which is not known to depend on sulfur supply, S-transferases or E1 enzymes with RHD domains. To better understand the functional diversification of an ancestral S-carrier into today’s members of the ubiquitin family, we therefore studied whether Urm1 dual-functions may be interlinked by comparing both tRNA thiolation and urmylation under *URM1* pathway inactivating conditions 36,38. Here we show that similar to heat-induced tRNA thiolation defects, Ahp1 urmylation in yeast is suppressed at 39°C. Moreover, as is the case with tRNA thiolation, Ahp1 urmylation is highly responsive to sulfur availability and requires the S-relay system that is dedicated for proper tRNA thiolation (via Urm1-COSH formation). In line with this, Urm1 functions in tRNA thiolation and urmylation depend on the rhodanese-type S-transfer region RHD in Uba4 (crucial for Urm1-COSH formation). In sum, the two *URM1* pathway branches, tRNA thiolation and protein urmylation, are chemically linked through sulfur supply, transfer and activation by the ubiquitin-like modifier system Uba4•Urm1.

## RESULTS

### Protein urmylation and tRNA thiolation are both thermosensitive 

**Figure 2:**
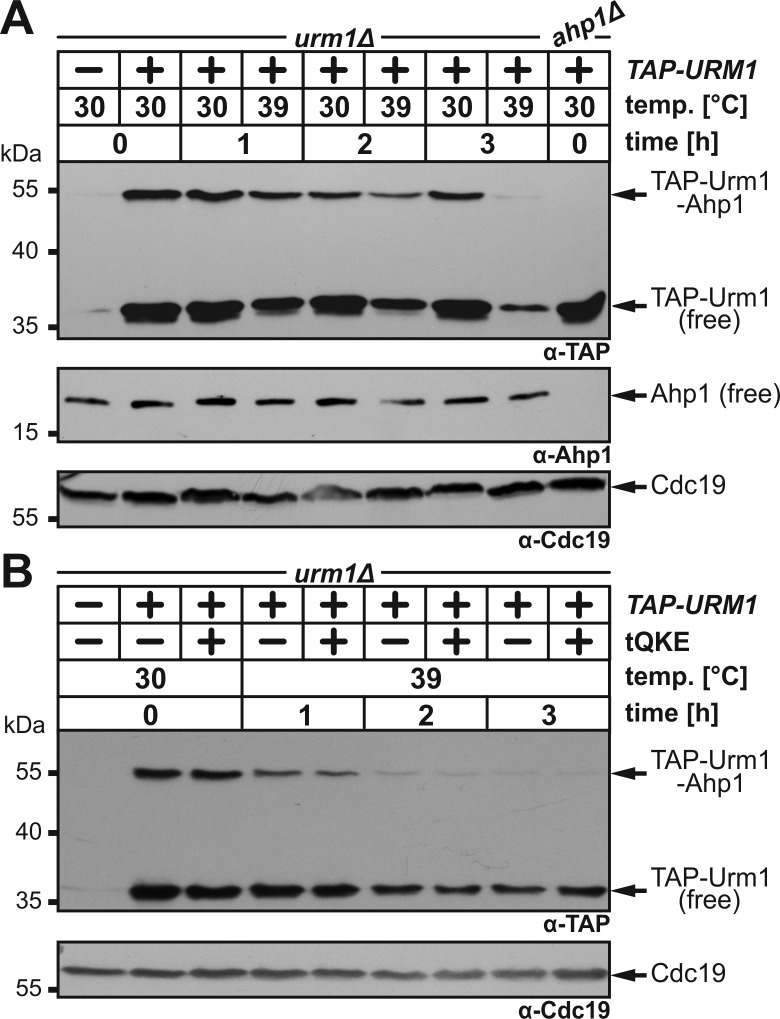
FIGURE 2: Overexpression of tRNAs subject to Urm1-dependent U34 thiolation fails to suppress loss of Ahp1 urmylation at 39°C. **(A)** Urmylation of Ahp1 is suppressed at 39°C. An *urm1*∆ strain expressing *TAP-URM1* was grown to logarithmic growth phase and split into two cultures prior to cultivation at 30°C or 39°C for three hours (h). Ahp1 urmylation analysis involved anti-TAP-based EMSA and immune blots with anti-Ahp1 to detect non-conjugated (free) Ahp1. Protein loading was controlled with anti-Cdc19 antibodies. **(B) **tRNA^Gln^, tRNA^Lys^ and tRNA^Glu^ (tQKE) overexpression cannot rescue defective Ahp1 urmylation at 39°C. Except for tQKE overexpression from multi-copy vector pQKE (Table S3), cell growth at 30°C or 39°C and urmylation analysis were as described in (A). Arrows (A, B) indicate the positions of non-conjugated (free) forms of TAP-Urm1 and Ahp1, as well as TAP-Urm1 conjugated to Ahp1 and loading control Cdc19.

Loss of tRNA thiolation causes heat-sensitive growth in *URM1 *pathway mutants 7,8,9 and recently, *URM1 *pathway inactivation at 37°C or 39°C was shown to trigger tRNA thiolation defects sufficient for growth inhibition 36,39,40,41. Hence, we studied the ability of Urm1 to engage in urmylation at temperatures restrictive for tRNA thiolation. To do so, we used a yeast strain that expresses a TAP-tagged Urm1 fusion (~35 kDa) previously shown to conjugate to proteins such as Ahp1 and Uba4 11. *TAP-URM1* cells were grown to logarithmic growth phase at 30°C and split into two cultures. One was kept at 30°C, the other shifted to 39°C and both cultivated for three hours prior to protein urmylation analysis. Using electrophoretic mobility shift assays (EMSA) based on anti-TAP Western blots 11, we detected at 30°C non-conjugated TAP-Urm1 (~35 kDa) and a prominent up-shifted (~55 kDa) TAP signal (Fig. 2A). We confirmed this is an Ahp1•TAP-Urm1 conjugate 11 by showing that it did not form in *ahp1*∆ mutants (Fig. 2A) and that it was further up-shifted when tagged in *AHP1-c-myc* cells (Fig. S1). At 39°C, however, formation of Ahp1•TAP-Urm1 conjugates gradually declined over time and was almost absent after three hours (Fig. 2A). Similarly, but less pronounced, the abundance of free TAP-Urm1 decreased over time, which contrasts with stable forms of unconjugated Ahp1 at 39°C (Fig. 2A). Our data thus indicate that rather than correlating with an unstable target, loss of Ahp1 urmylation at 39°C is likely due to a fragile Urm1 modifier itself.

tRNA thiolation and translation defects in *URM1 *pathway mutants cause phenotypes that can be rescued by overexpressing tRNAs normally undergoing mcm^5^s^2^U34 modifications 8,15,25,42,43. When higher-than-normal levels of these tRNAs, i.e. tRNA^Gln^, tRNA^Lys^ and tRNA^Glu^ [tQKE], were produced from a multi-copy plasmid, they failed to suppress either the Ahp1 urmylation defects or the low Urm1 abundance at 39°C (Fig. 2B). This suggests it is not a translational defect suppressible by tRNAs which underlies heat-sensitive urmylation. In support of this, we observed that translation inhibition by cycloheximide had no effect on Urm1 levels (Fig. S2). With previous data showing proteins required for S-transfer (Tum1) and Urm1 activation (Uba4) are unstable, too 36,38,39, heat-sensitivity of the *URM1* pathway may thus be multifactorial. In sum, Urm1 instability alone or combined with S-transfer defects at 39°C appear to inactivate urmylation and as previously shown, tRNA thiolation.

### Sulfur supply and activation link tRNA thiolation and urmylation

**Figure 3:**
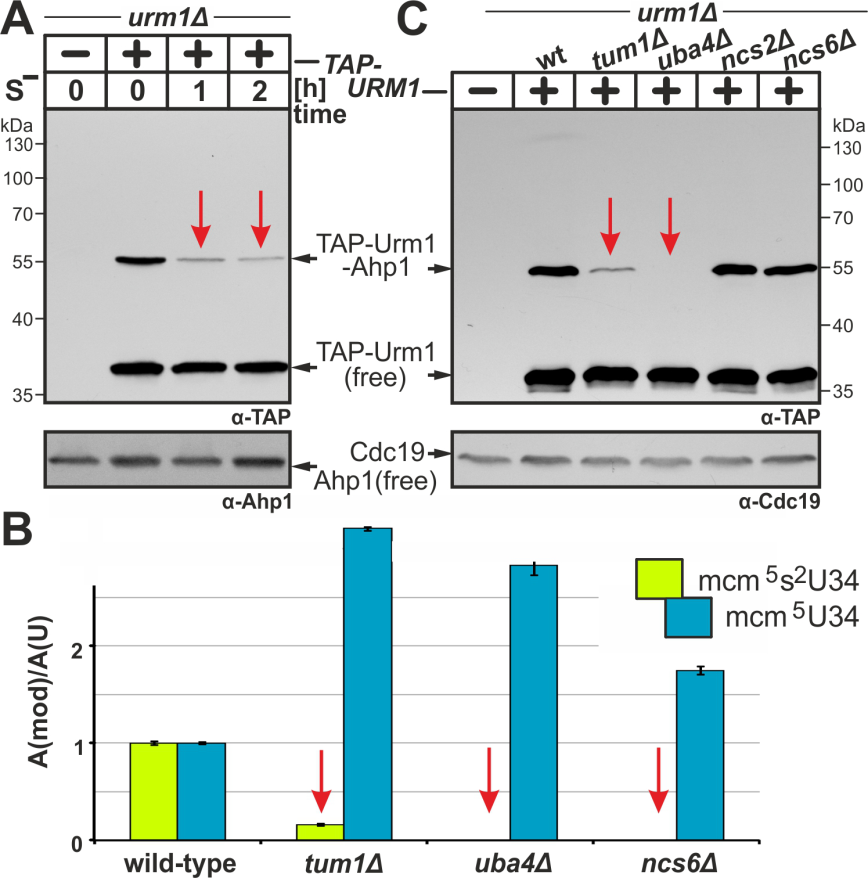
FIGURE 3: Sulfur supply, transfer and activation are critical for the tRNA thiolation and urmylation branches of the *URM1* pathway. **(A)** Urmylation is sulfur sensitive. *TAP-URM1* expressing cells (see Fig. 2) were grown in Met containing media prior to S-starvation (S^-^) for two hours (h) in media without Met. Analysis of Ahp1 urmylation was as described (see Fig. 2). **(B)** LC-MS/MS-based tRNA (thio)modification analysis of modified U34 nucleoside content in tRNAs from wild-type and indicated *URM1* pathway mutants. In each case, the modified nucleoside signal was normalized using total uridine content to allow comparison of the different samples. Inset: mcm^5^ (blue) and mcm^5^s^2^ (green) modifications at U34. **(C)** Urmylation of Ahp1 is normal in thiolase-minus (*ncs2*∆, *ncs6*∆) cells but drastically reduced in the absence of S-transferase Tum1 (*tum1*∆). Protein extracts from *TAP-URM1* expressors of the indicated backgrounds (i.e. wild-type (wt) and *URM1* pathway mutants) were subjected to Ahp1 urmylation assays as described (see Fig. 2). Non-conjugated (free) forms of TAP-Urm1 and Ahp1 as well as TAP-Urm1 conjugated to Ahp1 and loading control Cdc19 are indicated by arrows (A, C).

Under conditions of methionine starvation, sulfur consuming pathways including mcm^5^s^2^U34 modifications, which require Urm1, Elongator and S-adenosyl-L-methionine, have been shown to dramatically decline in yeast 38. This reinforces that sulfur supply and activation in form of Urm1-COSH is critical for tRNA thiolation 7,8,9. Hence, we compared sulfur dependency between the two *URM1* pathway branches, i.e. protein urmylation and tRNA thiolation, by examining the effects of starvation for the sulfur amino acid methionine (Met). *TAP-URM1* cells were shifted from Met-containing to Met-free media, and urmylation was analyzed by EMSA. In the presence of Met, we detected free TAP-Urm1 and the prominent Ahp1 conjugate (~55 kDa) (Fig. 3A). Interestingly, while free TAP-Urm1 and non-conjugated Ahp1 remained stable irrespective of sulfur supply, urmylation of Ahp1 by TAP-Urm1 dramatically declined during S-starvation (Fig. 3A). Thus sulfur depletion specifically suppresses Urm1 conjugation indicating that by analogy with tRNA thiolation, protein urmylation is also sensitive to sulfur supply.

To study whether the sulfur relay system implicated in the S-donor role of Urm1 for tRNA thiolation 7,8,9 also contributes to urmylation, we compared both *URM1 *pathway branches in mutants with appropriate genetic defects (Fig. 3B, C). Here, loss of Urm1 activator Uba4 abolished Ahp1 urmylation in *uba4*∆ cells (Fig. 3C) which is consistent with LC-MS/MS analysis showing loss of mcm^5^s^2^U34 modifications and increased mcm^5^U34 levels in tRNAs due to unaltered Elongator activity (Fig. 3B) 24,38,43. While thiolase-minus *ncs6*∆ cells produced U34 profiles similar to *uba4*∆ (Fig. 3B), both *ncs2*∆ and *ncs6*∆ mutants maintained wild-type Ahp1 urmylation levels (Fig. 3C). This agrees with previous data showing urmylation occurs in the absence of tRNA thiolation 4,5. These read-outs contrast sharply with those of *tum1*∆ cells lacking S-transferase Tum1 22,45. As revealed by LC-MS/MS and EMSA, they are significantly reduced in tRNA thiolation and Ahp1 urmylation (Fig. 3B, C). Indeed mcm^5^s^2^U34 modifications in *tum1*∆ cells declined to ~16% of wild-type levels (Fig. 3B) indicating that S-transfer via Tum1 is critical for proper tRNA thiolation and Ahp1 urmylation.

EMSA exposed for longer times (Fig. S3) showed urmylation of proteins other than Ahp1 is also affected in *tum1*∆ cells stressing the importance of S-transfer for Urm1 conjugation. Recently, yeast Uba4 and its human homolog (hUBA4/MOCS3) have been reported to undergo urmylation themselves 6,11, so we studied Uba4 urmylation in a *tum1*∆ strain that co-expressed c-Myc-tagged Uba4 and HA-marked Urm1. EMSA based on immune blots with anti-HA- and anti-c-Myc-antibodies allowed detection of Uba4•Urm1 conjugates (~130 kDa doublet band) (Fig. S4). Intriguingly, Uba4 urmylation was not sensitive to *TUM1* deletion (Fig. S4) and disruption of *NCS2*, *NCS6* or *AHP1* also had no effect on Urm1 conjugation to Uba4. To sum up, our data show that protein urmylation (except for Uba4) depends on sulfur supply and on the sulfur relay system (Nfs1, Tum1, Uba4) that contributes to the S-donor role for Urm1 in tRNA thiolation. Hence, S-transfer and Urm1 thiocarboxylation apparently link tRNA thiolation and urmylation to each other.

### tRNA thiolation and urmylation are linked by catalytic Cys residues in Uba4

**Figure 4:**
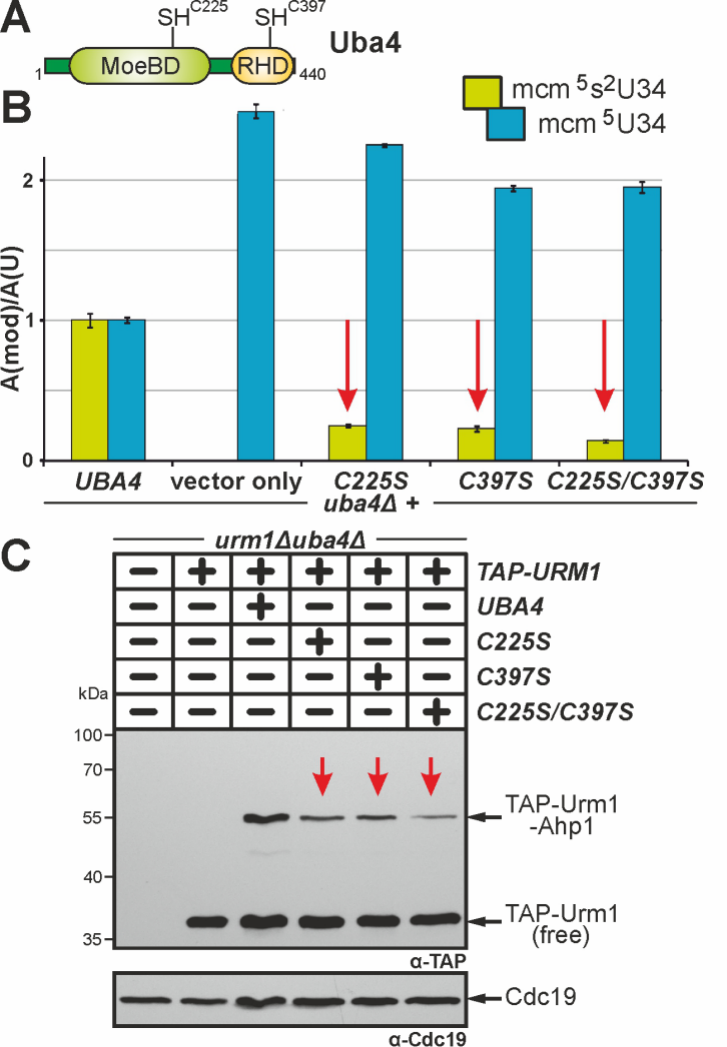
FIGURE 4: Cys225 and Cys397 are critical but not essential for Uba4 to activate Urm1 functions in tRNA thiolation and urmylation. **(A)** Uba4 scheme depicting MoeBD and RHD regions and the positions of Cys225 and Cys397. **(B)** tRNA (thio)modification analysis via LC-MS/MS (see Fig. 3) reveals Cys225 and/or Cys397 substitution mutants significantly interfere (red arrows) with formation of the mcm^5^s^2^U34 modification. **(C)** Ahp1 urmylation is strongly (red arrows) affected by the C225S, C397S and C225S/C397S mutants. Urmylation assays (see Fig. 2) involved anti-TAP-based EMSA to detect non-conjugated (free) TAP-Urm1 and TAP-Urm1•Ahp1 conjugates. Protein loading was controlled with anti-Cdc19 antibodies.

Uba4 maintains two domains (MoeBD; RHD) with catalytic cysteines (C225; C397) (Fig. 4A). This organisation is peculiar since no E1 or other E1-like enzyme carries rhodanese-like domains (RHD) typical of S-transfer proteins such as Tum1 21,45. Since sulfur transfer to Uba4 via Tum1 is critical for Urm1 activation, we revisited C225 and C397 and examined their contributions to tRNA thiolation and urmylation. Previously, loss of function phenotypes were ascribed to cysteine ablative alanine (C225A or C397A) mutations and some of these were reported to lead to unstable proteins 3,6,8,9,18.

Here, we generated single and double cysteine mimicking serine substitutions (C225S, C397S, C225S/C397S) that in striking contrast to the ablative ones differentially affected Uba4 functionality (Fig. 4). Using LC-MS/MS to compare thiolated (mcm^5^s^2^U34) and non-thiolated (mcm^5^U34) tRNA modifications between *UBA4* wild-type and *uba4*∆ cells, we reconfirmed (Fig. 3B) loss of U34 thiolation and mcm^5^U34 accumulation in the latter background (Fig. 4B). This agrees with previous data showing Uba4 is essential for tRNA thiolation 7,9,25,38,43. Intriguingly, rather than eliminating Uba4 function, C225S or C397S partially restored U34 thiolation in *uba4*∆ cells with mcm^5^s^2^U34 levels (25%: C225S; 23%: C397S) significantly reduced compared to wild-type (100%: *UBA4*) (Fig. 4B). Also, LC-MS/MS revealed mcm^5^s^2^U34 formation became further reduced in the double mutant (14%: C225S/C397S) (Fig. 4B) meaning that in combination, the two substitutions are additive and synthetically enhance the tRNA thiolation defects of the single mutants alone.

To prove these mcm^5^s^2^U34 defects are phenotypically relevant, we introduced the Cys substitutions into *uba4*∆ reporter cells with no functional Elongator (*elp3*∆) or Deg1 (*deg1*∆). In addition to loss of U34 thiolation, both reporter strains lack other tRNA modifications: Elp3 (as part of Elongator) cooperates with Uba4•Urm1 in mcm^5^s^2^U34 formation and Deg1 is necessary for pseudouridine synthesis at tRNA positions 38 and 39 46,47. The combined tRNA modifications defects in the two reporter strains result in thermosensitive growth that can be rescued by tRNA overexpression implying the phenotype is due to improper tRNA function 41,43,44. Hence, suppression of thermosensitivity by Uba4 rescues the consequences of the tRNA thiolation defects (Fig. S5). Using this assay diagnostic for tRNA thiolation, we found that while C225S and C397S alone partially rescued thermosensitive growth of the *elp3*∆*uba4*∆ and *deg1*∆*uba4*∆ reporter strains, the double mutant C225S/C397S only weakly suppressed *deg1*∆*uba4*∆ cells (Fig. S5). So, C225S, C397S and C225S/C397S progressively reduce the capacity of Uba4 to restore growth and tRNA thiolation, a notion congruent with their differential U34 modification profiles (Fig. 4B).

Using EMSA we next compared urmylation in the Cys substitution mutants to wild-type and found the single ones (C225S, C397S) were clearly reduced in TAP-Urm1•Ahp1 conjugate formation (Fig. 4C). Again, in the C225S/C397S double mutant, defective urmylation was even further aggravated compared to each mutant alone but, importantly, not entirely abolished (Fig. 4C). Together, these urmylation assays go hand-in-hand with the tRNA thiolation profiles (Fig. 4B) and indicate that the Cys residues in the MoeBD (C225) and RHD (C397) regions of Uba4 overlap in function and collectively, contribute to full Uba4 functionality for proper tRNA thiolation and urmylation.

### The rhodanese domain (RHD) in Uba4 links tRNA thiolation and urmylation

**Figure 5:**
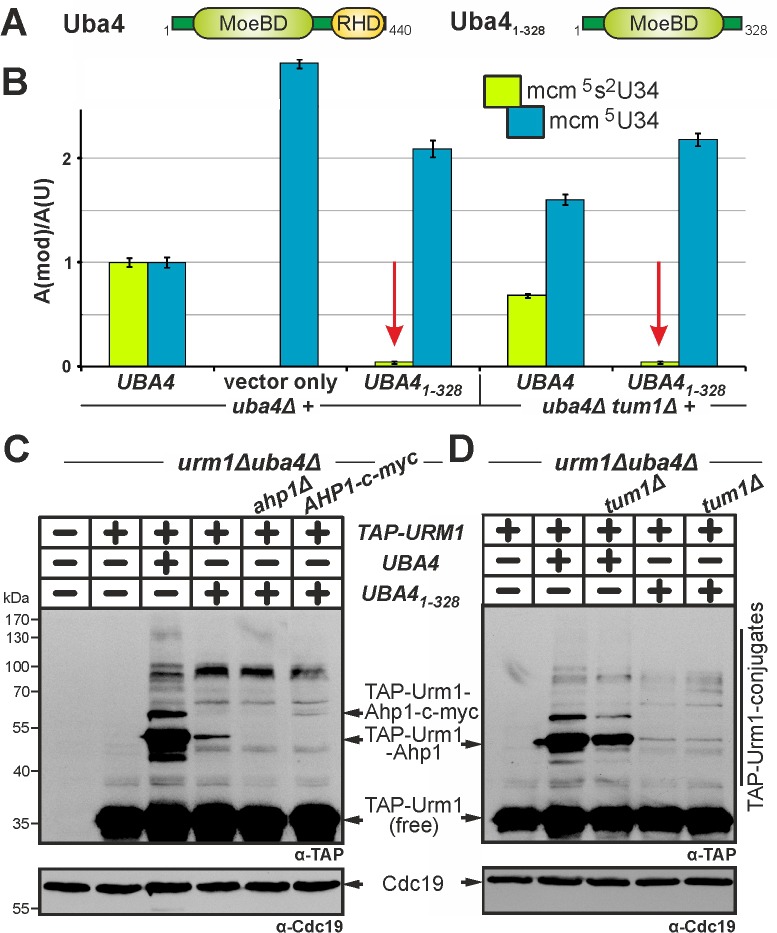
FIGURE 5: Low residual tRNA thiolation and urmylation without the RHD on Uba4 is independent of sulfur transfer by Tum1. **(A)** Scheme depicting Uba4_1 328_, which lacks the RHD region. **(B)** LC-MS/MS-based analysis (see Fig. 3) reveals Uba4_1 328_ is severely compromised in tRNA thiolation (red arrows) with the remaining residual (~4%) levels of mcm^5^s^2^-modified U34 insensitive to* TUM1* gene function. **(C, D)** Urmylation of proteins including Ahp1 is strongly suppressed in the absence of the RHD. Urmylation assays on protein extracts from the indicated genetic backgrounds involved anti-TAP-based EMSA (see Fig. 2) and immune blots to detect non-conjugated (free) TAP-Urm1 and TAP-Urm1 conjugates (C, D) including urmylated forms of wild-type (C, D) and c-myc-tagged Ahp1 (D). Protein loading (C, D) was controlled with anti-Cdc19 Western blots. Note that as is the case with very weak Tum1 insensitive tRNA thiolation (B), low residual levels of urmylation with Uba4_1 328_ are also unaltered in the *tum1*Δ mutant (D).

Having shown above that S-transfer is critical for Urm1 activation by Uba4 and that thiol-active cysteines are important to do so, we next focused on the role of the RHD, a rhodanese-like region in Uba4 with sulfur acceptor activity 18,23. Upon RHD removal from Uba4 (Fig. 5A), we examined the ability of the resulting truncation (Uba4_1-328_) to mediate tRNA thiolation and urmylation in *uba4*∆ cells. LC-MS/MS analysis showed that mcm^5^s^2^U34 modifications in tRNA anticodons from *UBA4_1-328_* cells dramatically declined down to ~4% of *UBA4 *wild-type levels (Fig. 5B). Markedly, these very low residual thiolation levels still partially suppressed the thermosensitive growth of the *elp3*∆*uba4*∆ and *deg1*∆*uba4*∆ reporter strains (Fig. S6). Thus, even without an RHD, Uba4_1-328_ apparently engages in residual Urm1 activation sufficient enough for low-level tRNA thiolation. Indeed, this notion also correlates with EMSA showing that urmylation including Urm1 conjugation to Ahp1 was drastically affected in *UBA4_1-328_* cells with low-level of Ahp1•TAP-Urm1 conjugates detectable after longer exposure times (Fig. 5C). Thus, our results identify the RHD region on Uba4 as the main contributor to Urm1 dual-functions in tRNA thiolation and urmylation.

When we tried to rescue *UBA4_1-328_* cells by co-expressing *UBA4_329-440_* (encoding the RHD alone), we were not able to improve tRNA thiolation based on its failure to rescue *deg1*Δ*uba4*Δ cell growth and suppress Urm1 conjugation defects (Fig. S7). Together with our observation that *UBA4_329-440_* alone could not rescue defective tRNA thiolation or urmylation (Fig. S7) in *uba4*∆ cells, our data suggest that in order to enable Urm1 activation and thiocarboxylation, both Uba4 domains (MoeBD & RHD) need to be maintained in close proximity or on the same polypeptide rather than being provided separately. In sum, our results show that although catalytically critical for Uba4, the RHD on its own is non-functional. Probably, this explains why the MoeBD alone in Uba4_1-328_ has residual E1-like activity sufficient for very low-level tRNA thiolation and urmylation.

### Tum1 feeds into tRNA thiolation and urmylation through the RHD in Uba4

Our data above indicate tRNA thiolation and urmylation defects in *tum1*∆ cells correlate with reduced S-transfer to Uba4 and improper Urm1 thiocarboxylation. Hence, we asked whether loss of *TUM1* would affect low-level tRNA thiolation and urmylation observed in the absence of the RHD (Uba4_1-328_). As revealed by LC-MS/MS, *UBA4_1-328_* cells still allowed for residual tRNA thiolation in the absence of Tum1 (Fig. 5B). Importantly, the remaining low mcm^5^s^2^U34 levels (~4% in relation to *UBA4 *wild-type) were similar, if not identical in both *TUM1 *and *tum1*∆ cells (Fig. 5B). To support this result, which points towards residual tRNA thiolation independent of *TUM1*, we investigated whether an additional *tum1*∆ null-allele affects the partial suppression of *elp3*∆*uba4*∆ or *deg1*∆*uba4*∆ growth by *UBA4_1-328_* (Fig. S6). If Tum1 accounted for residual activity of Uba4_1-328_, partial suppression may be abolished by *TUM1* gene deletion. However, the phenotypic assays clearly demonstrate the opposite: a *tum1*∆ null-allele has no effect on partial suppression of *elp3*∆*uba4*∆ or *deg1*∆*uba4*∆ cell growth by *UBA4_1-328_* (Fig. S6). Similarly, weak protein urmylation typical of *UBA4_1-328 _*cells was not altered in tandem with *tum1*∆ (Fig. 5D) indicating no additive effect of reduced S-transfer onto residual performance of Uba4_1-328_. This shows low residual levels of tRNA thiolation and urmylation in the absence of RHD are insensitive to Tum1, suggesting that Tum1-dependent S-transfer for proper function of Uba4 operates mainly, if not entirely, through its RHD region.

## DISCUSSION 

Compared to ubiquitin and ubiquitin-like proteins, Urm1 activation differs by thiocarboxylation 18,19. Urm1-COSH generated this way provides sulfur for tRNA thiolation (Fig. 1) and possibly protein urmylation 6,18,23. Such shared need for Urm1-COSH suggests both Urm1 functions are linked. Hence, we reasoned that conditions inactivating tRNA thiolation 36,38,39,40,41 may also impact on urmylation. We show herein that cells grown at 39°C or starved for sulfur drastically suppress urmylation. At 39°C, loss of urmylation correlated with decreased levels of Urm1 itself, while S-starvation had no such effect (Fig. 2). This goes along with other *URM1 *pathway proteins (Tum1, Uba4, Ncs2, Ncs6) also reported to be unstable at higher temperatures 36,39. While instable thiolase (Ncs2•Ncs6) affects tRNA thiolation, lowering Tum1 and Uba4 activities will interfere with S-transfer and Urm1 activation. So, suppression of urmylation at 39°C may be due to fragile Urm1 alone or combined with improper Urm1 activation 18,19. *URM1 *pathway inactivation in S-starved cells (Fig. 3) is readily explained by the need of sulfur for Urm1-COSH formation 6,18,23. In sum, suppression of tRNA thiolation and urmylation at 39°C or upon S-depletion likely involves inappropriate Urm1 thiocarboxylation.

Down-regulation of tRNA thiolation was suggested to reduce translational competence and cell growth under sulfur limiting conditions which may be important to spare sulfur for other physiologically important processes 38. Since we show Urm1 conjugation depends on Urm1-COSH and hence qualifies itself as a sulfur *consuming* process, loss of urmylation upon S-depletion could help spare sulfur as well. Also, we cannot exclude that a decrease in urmylation affects the activity of protein(s) that Urm1 attaches to. Probably, this may help to adapt to conditions known to be *URM1 *pathway related, i.e. ROS, thermo-stress, S-depletion 6,11,17,38,39,40,41. However, with attachment of ubiquitin-like modifiers such as SUMO regulating the activity of their targets in ways different from Urm1 2,48,49,50, how precisely urmylation impacts on its targets is still not clear.

**Figure 6:**
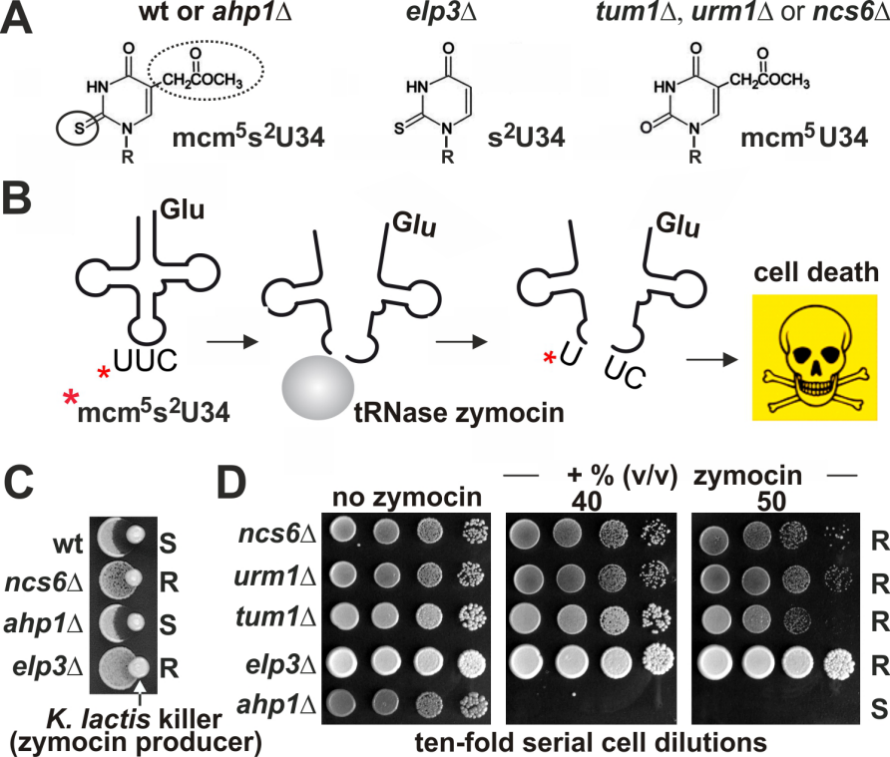
FIGURE 6: The role of Urm1 in tRNA thiolation, not in urmylation of Ahp1, drives tRNase toxicity. **(A)** Shown are wobble uridine (U34) modifications from different genetic backgrounds: 5-methoxycarbonyl-methyl-2-thiouridine (mcm^5^s^2^U-34), thiouridine (s^2^U34) and 5-methoxycarbonyl-methyl-uridine (mcm^5^U34). For simplicity, ‘R’ denotes ribose moieties. U34 thiolation (solid circle) requires S-transfer via Tum1, Urm1•Uba4 and thiolase Ncs2•Ncs6; mcm^5 ^side-chain (dotted circle) formation depends on Elongator 24,28. **(B)** The mcm^5^s^2^U34 modification (asterisk) in tRNA^Glu^_U*UC_ is efficiently cleaved by zymocin, a fungal tRNase lethal to *S. cerevisiae* cells (see C) 28,56,57. **(C, D)** U34 modification defects (*elp3*Δ, *tum1*Δ, *urm1*Δ, *ncs6*Δ) protect against zymocin and loss of Ahp1 urmylation (*ahp1*Δ) confers wild-type (wt) like sensitivity. Growth tests involved killer eclipse assays using *K. lactis* zymocin producer and the indicated *S. cerevisiae* tester strains (see C) or toxin plate assays with ten-fold serial dilutions of the indicated tester strains in absence (left panel) or presence (other panels) of different doses of zymocin purified from *K. lactis *(see D). ‘S’ and ‘R’ indicate toxin sensitive and resistant responses, respectively.

Our findings that it is the activated sulfur in Urm1-COSH, which links tRNA thiolation and urmylation, may provide support for a previous model 37 that sees urmylation as a means to restrict - rather than spare (see above) - sulfur flow by reducing the pool of free Urm1 available for S-transfer and tRNA thiolation. Consistent with this, Elongator mutants with *URM1* pathway related mcm^5^s^2^U34 modification defects (Fig. 1) allow urmylation to occur 5 and our data show thiolase mutants (*ncs2*∆, *ncs6*∆) maintain wild-type levels of Ahp1 urmylation (Fig. 3). To check if this relationship is reciprocal we asked whether tRNA thiolation is affected by loss of Ahp1 urmylation and profiled yeast growth inhibition by a fungal tRNase (zymocin) (Fig. 6A, B) that requires mcm^5^s^2^-modified U34 for lethal anticodon cleavage 28,51,52. While strains with defects in S-transfer (*tum1*Δ) and tRNA thiolation (*urm1*Δ, *ncs6*Δ) protected against zymocin (Fig. 6C,D) loss of Ahp1 urmylation (*ahp1*∆) could not. This indicates lethal anticodon cleavage due to proper tRNA thiolation occurs in the absence of Ahp1 urmylation. Together with our findings above, that tRNA thiolation is not required for Ahp1 urmylation, the two *URM1* pathway branches - albeit mechanistically linked through sulfur activation - seem to be functionally separated from (rather than dependent on) each other. So to our minds, an above scenario where sulfur flow for tRNA thiolation is kept in-check by Ahp1 urmylation seems unlikely. We cannot, however, exclude targets other than Ahp1 whose urmylation still may affect tRNA thiolation. In this context, it is noteworthy that hURM1 and Urm1-like proteins (SAMP, TtuB) form conjugates with human and prokaryal orthologs of yeast thiolase (CTU2, ATPBD3, NcsA, TtuA) 6,12,13. Whether this implies that S-transfer (via Urm1-COSH) for tRNA thiolation involves urmylation of components of the thiolase is unknown but may be an attractive twist to the above topic as it suggests the option of interdependence among the two *URM1* pathway branches. Although the S-donor role for tRNA thiolation has been demonstrated *in vitro* using human *URM1 *pathway players including hURM1 and CTU2 23, we are not aware of sulfur transfer during lysine-directed urmylation to targets that Urm1 attaches to in yeast or other organisms.

In support of our findings that tRNA thiolation and urmylation depend on sulfur (Fig. 3), both *URM1* pathway branches are significantly impaired in the absence of sulfur transferase Tum1. Interestingly, Tum1 seems not to contribute to urmylation of Uba4 suggesting this conjugation does not rely on S-transfer via Tum1 for Urm1-COSH formation (Fig. S4). Instead, an alternative mechanism, more similar to E1-like activation of ubiquitin-like modifiers, may be envisaged involving thioester bond formation prior to lysine-directed urmylation 21. Remarkably, SUMO, a ubiquitin-like modifier, SUMOylates the Uba2 subunit of its own E1 complex at Lys residue 236 and without any E2 assistance 53. Since E2/E3 urmylation enzymes are elusive, Uba4•Urm1 *auto-urmylation *comparable to Uba2•SUMO *auto-SUMOylation* may be plausible. Indeed, using site-directed *UBA4* mutagenesis, we identified Lys candidate residues (K122, K248) that differentially contribute to Uba4 or Ahp1 urmylation (Fig. S8).

In contrast to previous reports 3,9,18, we demonstrate here that Cys residues in the MoeBD (C225) and RHD (C397) domains of Uba4 are catalytically important for formation of Urm1-COSH yet not essential (Fig. 4). This is based on our findings that mimetic Ser substitutions alone or combined (C225S, C397S, C225S/C397S) cannot entirely abolish Uba4 functions but progressively reduce tRNA thiolation and urmylation to levels (14-25%) sufficient enough to form significant amounts of Urm1 conjugates and s^2^U34-modifed tRNA anticodons (Fig. 4). While it was shown that C397 can be persulfurated, which results in acyl-disulfide bond formation with Urm1 and subsequently in the release of Urm1-COSH 8,9,18,23, the role of C225 is unclear. C225 is analogous to active-site Cys residues in ubiquitin-like E1 enzymes but we are not aware it forms a thioester bond with Urm1 *in vivo*. Moreover, it is not essential for *in vitro* adenylation or thiocarboxylation of human URM1 by MOCS3/hUBA4 18. Hence, a potential role for C225 in the reductive cleavage of the acyl-disulfide bond between MOCS3 and hURM1 was proposed 18. Our data showing residual sulfur transfer with C225S alone or combined with C397S (Fig. 4) may indicate the presence of another Cys residue capable of reductive cleavage and Urm1-COSH release. An alternative cysteine present in Uba4 or provided by yet another protein might also explain why the C397S mutant allowed, albeit at significantly reduced efficiencies, S-transfer for tRNA thiolation and urmylation (Fig. 4). Residual low-level tRNA thiolation and urmylation are even more compromised with Uba4_1-328_, the truncation lacking the RHD (including C397) and importantly, uncoupled from Tum1 (Fig. 5). This suggests an alternative sulfur mode of transfer which either involves a Cys residue other than C225 in the MoeBD motif of Uba4 or an S-donor distinct from Tum1 (Fig. S9). The latter (if existent) may be identified among candidates with assigned or cryptic RHDs of the rhodanese protein family 54,55.

## Materials and Methods

### Yeast strains, general methods and plasmid constructions

Growth of yeast strains (Table S1) was in routine YPD or SC media 56 for 3 days, and thermosensitivity was assayed on YPD media at 34°C, 35°C or 39°C. Table S2 lists primers used for PCR-based protocols 57,58 to introduce site-specific *UBA4 *mutations or generate and diagnose gene deletions. Uba4 cysteine to serine substitutions C225S, C397S and C225S/C397S carried on plasmids pAJ64, pAJ65 and pAJ69, respectively (Table S3) were generated by PCR-based site-directed mutagenesis using a previously described protocol 59. Correctness of each mutation was confirmed by Sanger-based DNA sequencing. In analogy, site-directed *UBA4* mutagenesis of lysine residues (K122, K132, K156, K248) in the MoeBD of Uba4 resulted in arginine substitutions (Table S2). For *UBA4_1-328_* expression in yeast, the ORF coding for the N-terminal MoeB-like domain (MoeBD) of Uba4 alone was PCR-amplified from template plasmid pAJ16 11. Using flanking *Not*I and *Nde*I restriction sites, the *UBA4_1-328_*-construct was then cloned into vector pAJ16, resulting in generation of pAJ82 (Table S3). Construction of plasmid pAJ113 (Table S3) for expression of the rhodanese-type domain (RHD) of Uba4 alone started with PCR-amplification of the *UBA4_329-440_*-T*_CYC1_*-fragment from template plasmid pAJ16 11. This fragment was subcloned into *Nde*I- and *Sac*I-digested plasmid pAJ16, giving rise to a *UBA4_329-440_*-T*_CYC1_*-fragment, which was finally moved to yeast single copy vector pRS423 60 restricted with *Bam*HI and *Sac*I. Transformation of yeast cells with PCR products or plasmids (Table S3) was done as previously described 61. Qualitative assays to monitor sensitivity or resistance of *S.* *cerevisiae *cells to growth inhibition by the zymocin tRNase toxin complex involved previously described killer eclipse bioassays 33. In more sensitive assays, growth performance of ten-fold serial dilutions of *S. cerevisiae *tester strains was monitored for 2-3 days at 30°C on YPD plates containing no toxin or 40-50% (v/v) partially purified zymocin 33. Both assays used *K. lactis *killer strain AWJ137 (Table S1) as zymocin producer.

### tRNA modification profiling

Total tRNA was isolated from yeast cultures and subjected to LC-MS/MS for tRNA anticodon modification analysis essentially as previously described 26,35,62. Identification of mcm^5^U34 or mcm^5^s^2^U34 peaks was according to Jüdes *et al.* 11. For intersample comparability of the detected modifications, the peak areas of the modified nucleosides, measured in triplicates, were normalized to the UV peak area of uridine.

### Urmylation studies using electrophoretic mobility shift assays (EMSA)

Urmylation studies were done essentially as described 11 with yeast grown in standard SC media at 30°C to an OD_600_ of ~1.0 56. To analyze whether Urm1 conjugation is affected by elevated temperatures, logarithmically growing yeast cells were shifted from 30°C to 39°C and sampled after 1-3 hours of incubation at 39°C. To monitor sulfur dependency of urmylation, methionine auxotrophic (*met15*Δ) cells in the background of BY4741 (Table S1) were pregrown to logarithmic growth phase in standard SC minimal media 56 containing the sulfur amino acid methionine (2 mg/ml), washed and further suspended in SC media without methionine as a sulfur source. Finally, cells were harvested after 1 and 2 hours of additional cultivation and broken open with a bead beater and lysed in a buffer (10 mM K-HEPES pH 7.0, 10 mM KCl, 1.5 mM MgCl_2_, 0.5 mM PMSF, 2 mM benzamidine) containing complete protease inhibitors (Roche) and 10 mM N-ethylmaleimide (NEM) as previously described 6,11. EMSA and Western blot analyses used PVDF membranes essentially as described 11. Detection of unconjugated Ahp1 in NEM-free samples used anti-Ahp1 serum 63 provided by Dr Kuge (Tohoku Pharmaceutical University, Japan). Protein loading was checked using anti-Cdc19 antibodies donated by Dr Thorner (University of California, USA).

## SUPPLEMENTAL MATERIAL

Click here for supplemental data file.

All supplemental data for this article are also available online at http://microbialcell.com/researcharticles/sulfur-transfer-and-activation-by-ubiquitin-like-modifier-system-uba4%E2%80%A2urm1-link-protein-urmylation-and-trna-thiolation-in-yeast/.
