# Supplementary material for: Sulfur transfer and activation by ubiquitin-like modifier system Uba4•Urm1 link protein urmylation and tRNA thiolation in yeast
Source: Microb Cell. 2016 Oct 24;3(11):554–64. doi: 10.15698/mic2016.11.539 (PMC5349211; doi:10.15698/mic2016.11.539)
Supplement: Supplementary file 1 [file mic-03-554-s01.pdf]

Supplemental Data

Sulfur transfer and activation by ubiquitin-like modifier system Uba4•Urm1 link protein urmylation and tRNA thiolation in yeast

André Jüdes[1], Alexander Bruch[1], Roland Klassen[1], Mark Helm[2] and Raffael Schaffrath[1]*

[1] Universität Kassel, Institut für Biologie, FG Mikrobiologie
Heinrich-Plett-Str. 40, 34132 Kassel, Germany

[2] Johannes Gutenberg Universität Mainz, Institut für Pharmazie und Biochemie
Staudinger Weg 5, 55128 Mainz, Germany

***** Corresponding author: eMail: schaffrath@uni-kassel.de
Phone: +49-561-804-4175
FAX: +49-561-804-4337

1. Supplementary Tables

Table S1. Yeast strains used in this study.

Strain	Genotype	Source
BY4741	*MATa his3Δ1 leu2Δ0 met15Δ0 ura3Δ0*	Euroscarf
Y01400	BY4741, *urm1Δ::kanMX4*	Euroscarf
Y01939	BY4741, *uba4Δ::kanMX4*	Euroscarf
Y02507	BY4741, *tum1Δ::kanMX4*	Euroscarf
Y07242	BY4741, *ncs2Δ::kanMX4*	Euroscarf
Y04577	BY4741, *ncs6Δ::kanMX4*	Euroscarf
Y02720	BY4741, *ahp1Δ::kanMX4*	Euroscarf
Y02742	BY4741, *elp3Δ::kanMX4*	Euroscarf
Y07270	BY4741, *deg1Δ::kanMX4*	Euroscarf
RK28	BY4741, *elp3Δ::kanMX4 uba4Δ::ScHIS3*	[1]
FEY14	BY4741, *urm1Δ::kanMX4 AHP1-c-myc::ScHIS3*	[2]
FEY15	BY4741, *urm1Δ::kanMX4 uba4Δ::natNT2*	[2]
FEY16	BY4741, *ahp1Δ::kanMX4 urm1Δ::ScHIS3*	[2]
FEY19	BY4741, *ncs2Δ::kanMX4 urm1Δ::SpHIS5*	this study
FEY20	BY4741, *ncs6Δ::kanMX4 urm1Δ::SpHIS5*	this study
FEY21	BY4741, *tum1Δ::kanMX4 urm1Δ::SpHIS5*	this study
FEY25	BY4741, *urm1Δ::kanMX4 uba4Δ::natNT2 ahp1Δ::SpHIS5*	[2]
FEY26	BY4741, *urm1Δ::kanMX4 uba4Δ::natNT2 AHP1-c-myc::ScHIS3*	[2]
FEY31	BY4741, *uba4Δ::kanMX4 deg1Δ::SpHIS5*	this study
FEY32	BY4741, *urm1Δ::kanMX4 uba4Δ::natNT2 tum1Δ::SpHIS5*	this study
FEY34	BY4741, *urm1Δ::kanMX4 uba4Δ::natNT2 ncs2Δ::SpHIS5*	this study
FEY35	BY4741, *urm1Δ::kanMX4 uba4Δ::natNT2 ncs6Δ::SpHIS5*	this study
FEY41	BY4741, *elp3Δ::kanMX4 uba4Δ::ScHIS3 tum1Δ::KlURA3*	this study
FEY49	BY4741, *uba4Δ::kanMX4 deg1Δ::SpHIS5 tum1Δ::KlURA3*	this study
FEY50	BY4741, *uba4Δ::kanMX4 deg1Δ::loxP*	this study
AWJ137	*Kluyveromyces lactis* zymocin producing killer strain	Lab stock

Table S2. Primers used in this study.

Primer	Sequence (5'-3')	Application
DEG1koF	ggtgcccacatgcaatctttactgccctactataacctcccttgacagctgaagcttcgtacgc	*DEG1* ko
DEG1koR	gaaatatagtcttcaaggttatattatacaggtttatatattattgcataggccactagtggatctg	*DEG1* ko
KO_NCS2_FW	tgctattgtccatccctatcctagttttaaaaatataattctatcaagttcagctgaagcttcgtacgc	*NCS2* ko
KO_NCS2_RV	taaataaataaatacataaccattggaatagcgaagcctttgacatttcagcataggccactagtggatctg	*NCS2* ko
KO_NCS6_FW	aaaattttggcgatgagacgatatggtaagagtaaagcaaaggaaccgtccagctgaagcttcgtacgc	*NCS6* ko
KO_NCS6_RV	tatattatattatgttacgctgcattcttctactgcgagctatatatatggcataggccactagtggatctg	*NCS6* ko
KO_TUM1_FW	acaatgaggacaaaagcataaagttgtgaagaaaattgcccatacattcacagctgaagcttcgtacgc	*TUM1* ko
KO_TUM1_RV	ttaatatatgtagctaaataaatcgacttgtcaagaatatatttctcttagcataggccactagtggatctg	*TUM1* ko
KO_URM1_FW	caatactgatttctgatactaaaacgagataggttaatagcaaaatcgggcagctgaagcttcgtacgc	*URM1* ko
KO_URM1_RV	ctttatatatatatatgtagctgcttcttaaaaattatttgctgctatttgcataggccactagtggatctg	*URM1* ko
UBA4_C225S_FW	ccaaatgccgtgacctcttcccaagaaggcggtgtgatag	*UBA4* SM
UBA4_C225S_RV	ctatcacaccgccttcttgggaagaggtcacggcatttgg	*UBA4* SM
UBA4_C397S_FW	cagtaatatagtgattctttcccgctacggtaacgactctc	*UBA4* SM
UBA4_C397S_RV	gagagtcgttaccgtagcgggaaagaatcactatattactg	*UBA4* SM
UBA4_K122R_FW	gagtcggaatgttgagatgtgagtcggccag	*UBA4* SM
UBA4_K122R_RV	ctggccgactcacatctcaacattccgactc	*UBA4* SM
UBA4_K132R_FW	gccaggcaatatatcacacgagactgaacccacacattaac	*UBA4* SM
UBA4_K132R_RV	gttaatgtgtgggttcagtctcgtgatatattgcctggc	*UBA4* SM
UBA4_K156R_FW	ccagtaatgcttttgacattttcagaggttacaattatatattagactg	*UBA4* SM
UBA4_K156_RV	cagtctaatatatataatgtaacctctgaaaatgtcaaaagcattactgg	*UBA4* SM
UBA4_K248R_FW	gatggctgtagaaactttgagacttatcctaggaatctaca	*UBA4* SM
UBA4_K248R_RV	gtgtagattcctaggataagtctcaaagtttctacagccatc	*UBA4* SM
UBA4_FW_NdeI	gggcatatgaatgactaccatctcgaggataccacgtctg	PC [1]
UBA4_RV_NotI	ggggcggccgccagtgtgatggatatctgcagaattcctagtccacactgattctctcatc	PC [1]
UBA4(329-440)_fw_NdeI	catatggcatttcagcgtatctacaagg	PC [2]
MOCS3/UBA4_rv_SacI	cccgagctcaaagccttcgagcgtccc	PC [2]

Abbreviations:

ko: knock-out
SM: site-directed mutagenesis
PC: plasmid construction of [1]pAJ82 or [2]pAJ113 (see Table S3).

Table S3. Plasmids used in this study.

Plasmid	Description	Reference
pRS423	2µ ori, *ScHIS3*	[3]
YCplac33	*ARS1-CEN4*, *ScURA3*	[4]
YCplac111	*ARS1-CEN4*, *ScLEU2*	[4]
YEplac195	2µ ori, *ScURA3*	[4]
pHA-URM1	*HA-URM1* cloned into pRS426$_{SmaI}$	[5]
pCB45	P*GAL1-TAP-URM1* cloned into YCplac33$_{HindIII/SalI}$	[2]
pAJ16	P_{ADH1}-*UBA4*-T_{CYC1} cloned into YCplac111$_{BamHI/SacI}$	[2]
pAJ52	P_{ADH1}-*UBA4-c-myc*-T_{CYC1} cloned into YCplac111$_{EcoRI/SacI}$	[2]
pAJ64	P_{ADH1}-*uba4-C225S*-T_{CYC1} cloned into YCplac111$_{BamHI/SacI}$	this study
pAJ65	P_{ADH1}-*uba4-C397S*-T_{CYC1} cloned into YCplac111$_{BamHI/SacI}$	this study
pAJ69	P_{ADH1}-*uba4-C225S/C397S*-T_{CYC1} cloned into YCplac111$_{BamHI/SacI}$	this study
pAJ82	$UBA4_{1-328}$ cloned into pAJ16$_{NotI/NdeI}$	this study
pAJ105	P_{ADH1}-*uba4-K122R-c-myc* cloned into YCplac111$_{EcoRI/SacI}$	this study
pAJ106	P_{ADH1}-*uba4-K132R-c-myc* cloned into YCplac111$_{EcoRI/SacI}$	this study
pAJ107	P_{ADH1}-*uba4-K248R-c-myc* cloned into YCplac111$_{EcoRI/SacI}$	this study
pAJ108	P_{ADH1}-*uba4-K156R-c-myc* cloned into YCplac111$_{EcoRI/SacI}$	this study
pQKE	tQ^{UUG} tK^{UUU} tE^{UUC} cloned into pRS425	[2]
pAJ113	P_{ADH1}-$UBA4_{329-440}$-T_{CYC1} cloned into pRS423$_{BamHI/SacI}$	this study

2. Supplementary Figures

Fig. S1:

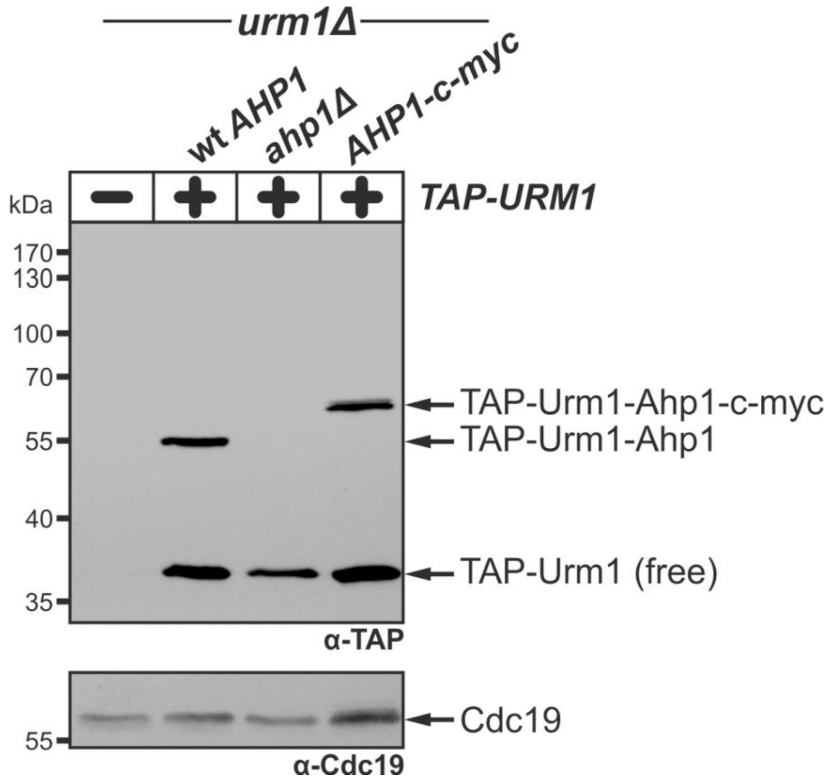

FIGURE S1: Peroxiredoxin Ahp1 is targeted for urmylation in yeast. Protein extracts from an *urm1*Δ reporter strain expressing *TAP-URM1* in the indicated genetic backgrounds, i.e. wild-type (wt) *AHP1*, *ahp1*Δ or *AHP1-c-myc*, were subjected to anti-TAP-based EMSA or immune blots with anti-Cdc19 antibodies (loading control). Arrows indicate the positions of non-conjugated (free) TAP-Urm1 as well as TAP-Urm1 conjugated to Ahp1 or Ahp1-c-myc, respectively.

Fig. S2:

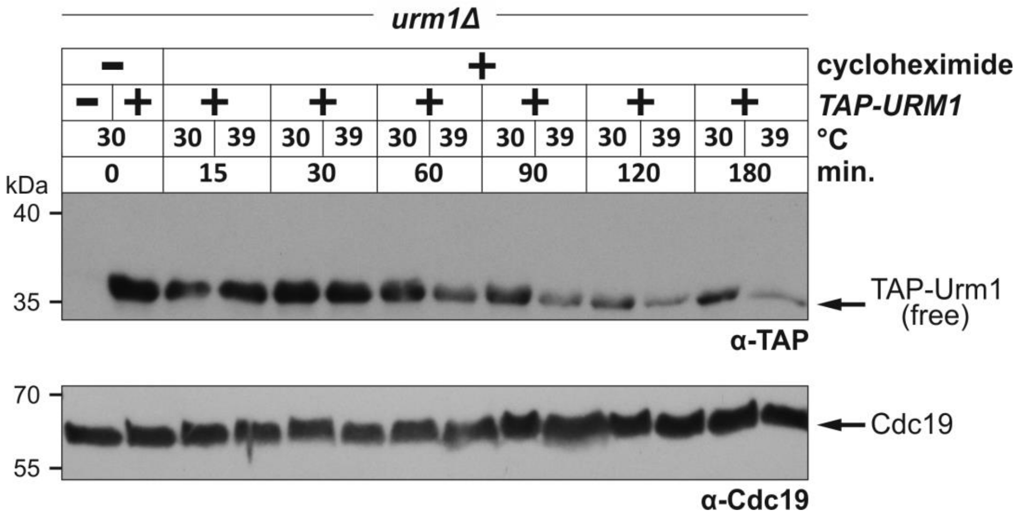

FIGURE S2: The decrease in Urm1 abundance at 39°C is independent of cycloheximide inhibition of translation. An *urm1*Δ strain expressing *TAP-URM1* was grown at 30° or 39°C for the indicated time in the absence (-) or presence (+) of 200 µg/ml cycloheximide. Upon protein extraction, Western blots (see Fig. S1) were used to compare protein stability and abundance between non-conjugated (free) TAP-Urm1 and pyruvate kinase (Cdc19) used as loading control.

Fig. S3:

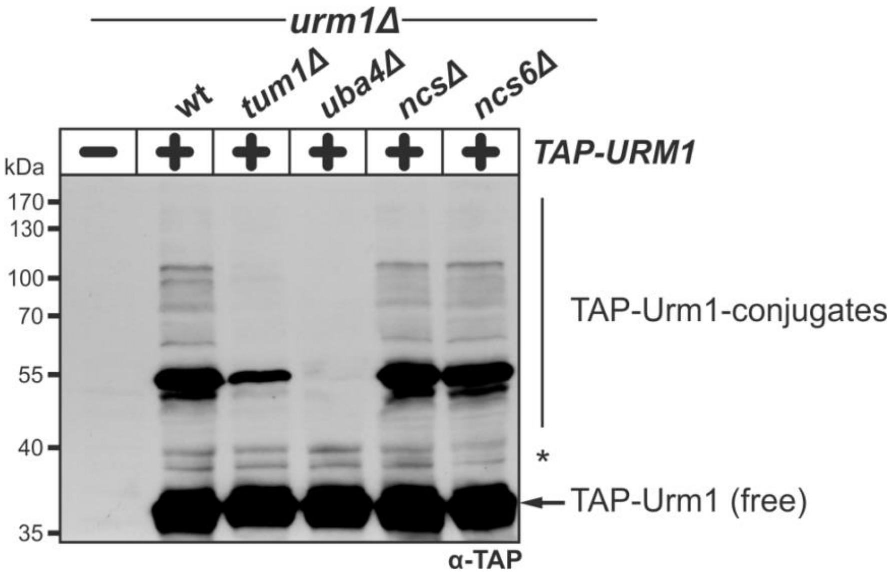

FIGURE S3: Global urmylation efficiency is reduced in a *tum1*Δ mutant. Protein extracts from an *urm1*Δ strain expressing *TAP-URM1* were obtained from wild-type (wt), *tum1*Δ, *uba4*Δ, *ncs2*Δ or *ncs6*Δ strain backgrounds and subjected to Urm1 conjugation studies (see Fig. S1). Unspecific TAP-Urm1 dependent signals are marked (*).

Fig. S4:

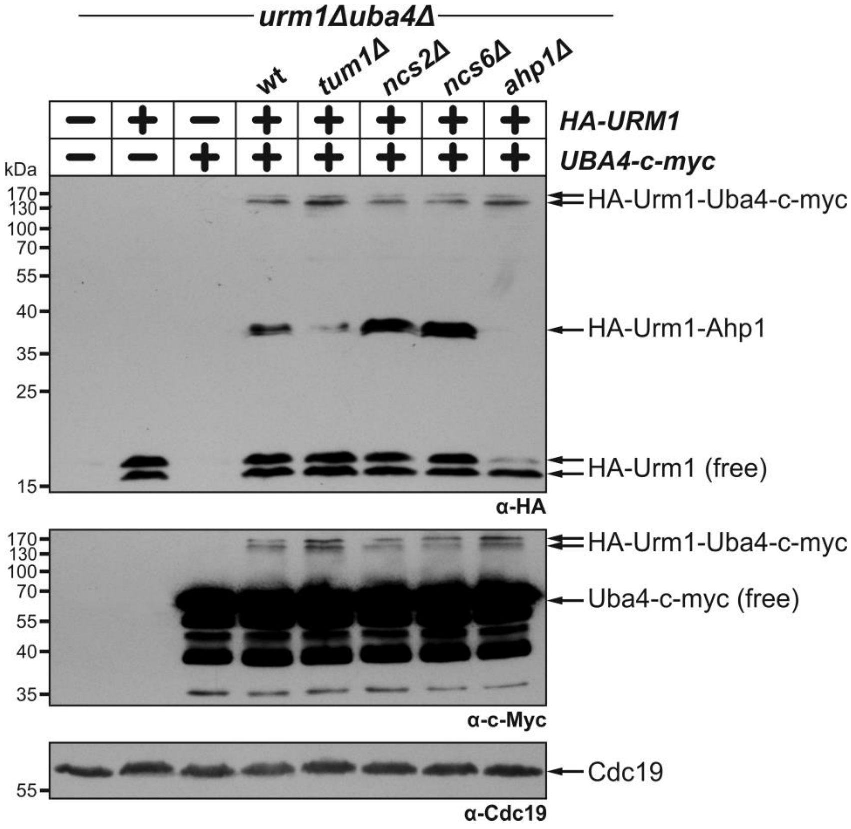

FIGURE S4: Unlike Ahp1, urmylation of Uba4 seems not to be affected by lack of sulfur transferase Tum1. Conjugation of HA-tagged Urm1 to c-myc-marked Uba4 was analyzed in wild-type (wt), *tum1*Δ, *ncs2*Δ, *ncs6*Δ or *ahp1*Δ backgrounds using EMSA (see Fig. S1) and Western blots specific for HA (Urm1), c-myc (Uba4) and Cdc19 (loading control). Arrows indicate non-conjugated (free) forms of c-myc-tagged Uba4 and HA-marked Urm1 as well as Ahp1 and c-myc-tagged Uba4 each conjugated to HA-Urm1.

Fig. S5:

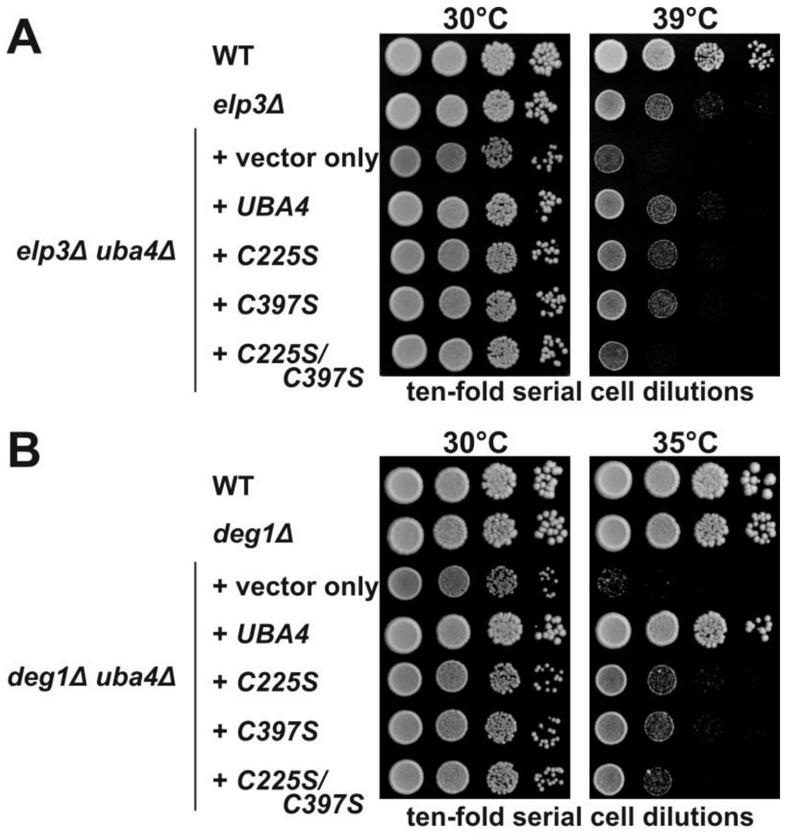

FIGURE S5: Thermosensitivity of *elp3*Δ*uba4*Δ or *deg1*Δ*uba4*Δ strains is partially rescued by Cys to Ser substitution mutations (C225S, C397S and C225S/C397S) in Uba4. Ten-fold serial dilutions of *elp3*Δ*uba4*Δ (**A**) or *deg1*Δ*uba4*Δ (**B**) reporter strains transformed with the indicated *UBA4* wild-type or mutant alleles were cultivated at 30°C (**A, B**), 35°C (**B**) or 39°C (**A**) for 3 days on YPD medium and compared to single *elp3*Δ (**A**) or *deg1*Δ (**B**) mutants alone and wild-type (WT) cells.

Fig. S6:

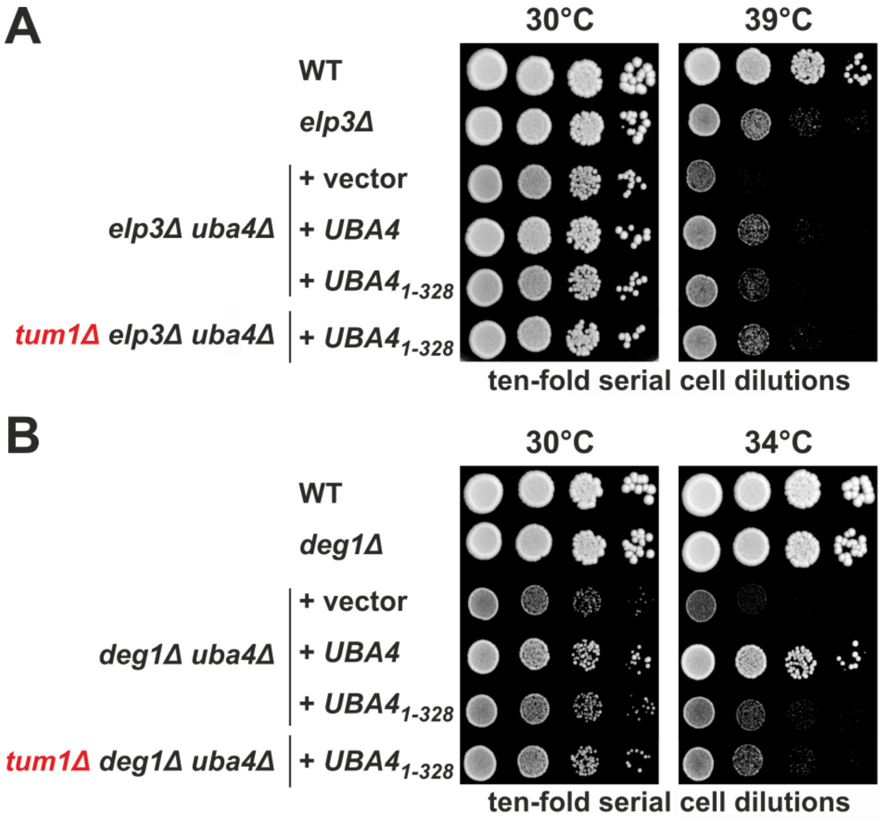

FIGURE S6: Temperature sensitivity of *elp3*Δ*uba4*Δ or *deg1*Δ*uba4*Δ reporter strains is slightly suppressed by Uba4$_{1\text{-}328}$ and in a fashion independent of *TUM1* gene function. Ten-fold serial dilutions of *elp3*Δ*uba4*Δ (**A**) or *deg1*Δ*uba4*Δ (**B**) reporter strains transformed with the indicated *UBA4* wild-type or mutant alleles or carrying an additional *TUM1* gene deletion (*tum1*Δ) were cultivated at 30°C (**A, B**), 34°C (**B**) or 39°C (**A**) for 3 days on YPD medium and compared to single *elp3*Δ (**A**) or *deg1*Δ (**B**) mutants alone and wild-type (WT) cells.

Fig. S7:

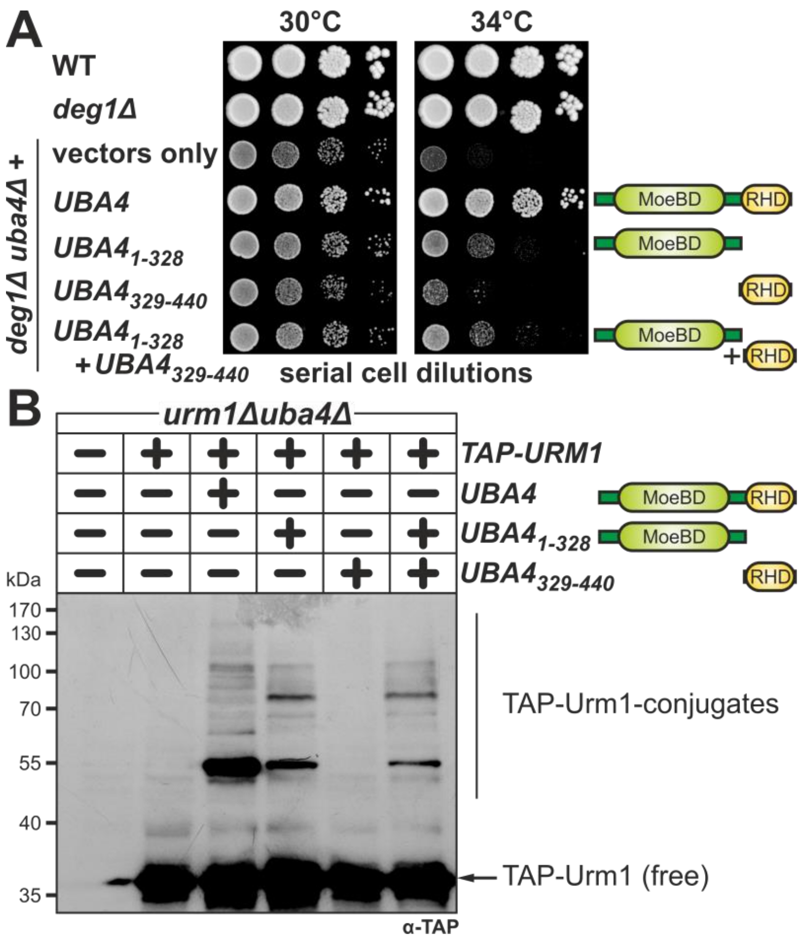

FIGURE S7: Proper Uba4 functions in tRNA thiolation and urmylation are not sustained upon co-expression of its individual MoeBD ($Uba4_{1\text{-}328}$) and RHD ($Uba4_{329\text{-}440}$) domains. (A) Expression of $Uba4_{329\text{-}440}$ alone or together with $Uba4_{1\text{-}328}$ fail to complement tRNA thiolation defects. The indicated *UBA4* alleles or vector controls were introduced into the *deg1*Δ*uba4*Δ reporter strain. Following cultivation at 30°C or 34°C (see Fig. S6B) thermosensitivity was compared between the transformants, the single *deg1*Δ mutant alone and wild-type (WT) cells. **(B)** RHD ($Uba4_{329\text{-}440}$) and MoeBD ($Uba4_{1\text{-}328}$) co-expression cannot rescue protein urmylation defects, and RHD expression alone copies *uba4*Δ cells lacking urmylation. Urmylation studies used EMSA based on anti-TAP Western blots (see Fig. S1). Non-conjugated (free) TAP-Urm1 and different TAP-Urm1 conjugates are indicated.

Fig. S8:

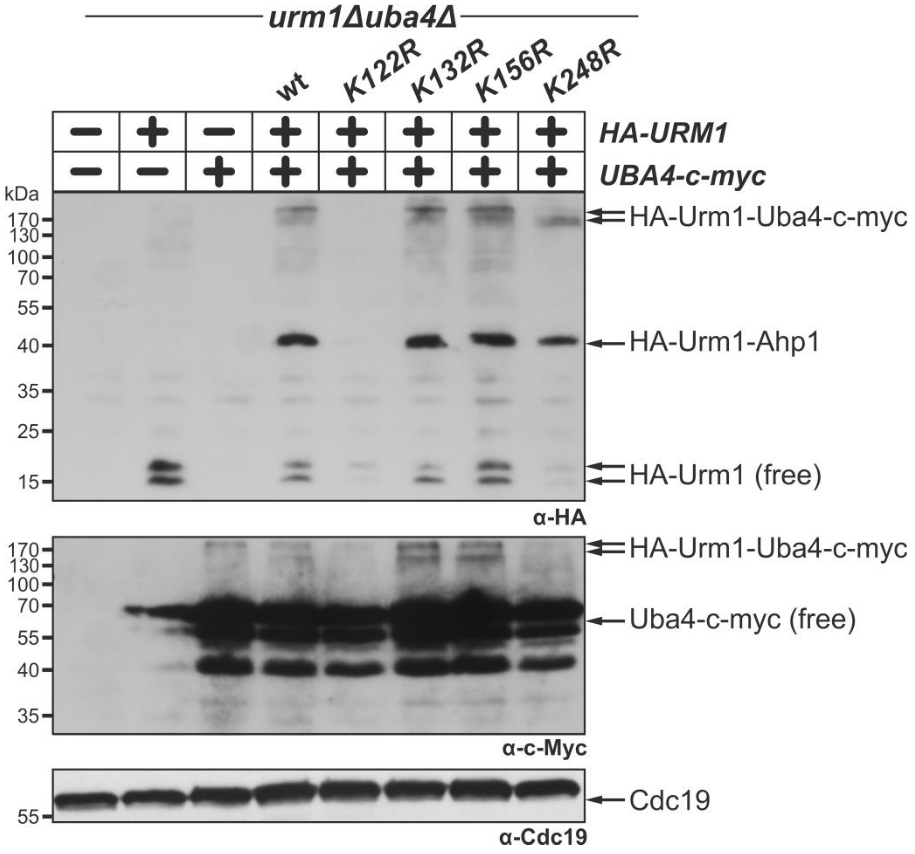

FIGURE S8: Site-directed mutagenesis of the MoeBD region in Uba4 identifies candidate Lys residues critical for urmylation of Ahp1 and/or Uba4 itself. Conjugation of HA-tagged Urm1 to Ahp1 and c-myc-marked Uba4 was analyzed in *uba4*Δ mutant cells carrying wild-type (wt) *UBA4* allele and the indicated Uba4 Lys (K) to Arg (R) substitution mutations. Urmylation was assessed by EMSAs (see Fig. S4) using Western blots specific for HA (Urm1), c-Myc (Uba4) and Cdc19 (loading control). Arrows indicate non-conjugated (free) forms of Uba4-c-myc and HA-Urm1 as well as HA-Urm1 conjugated to Ahp1 and c-myc-tagged Uba4, respectively.

Fig. S9:

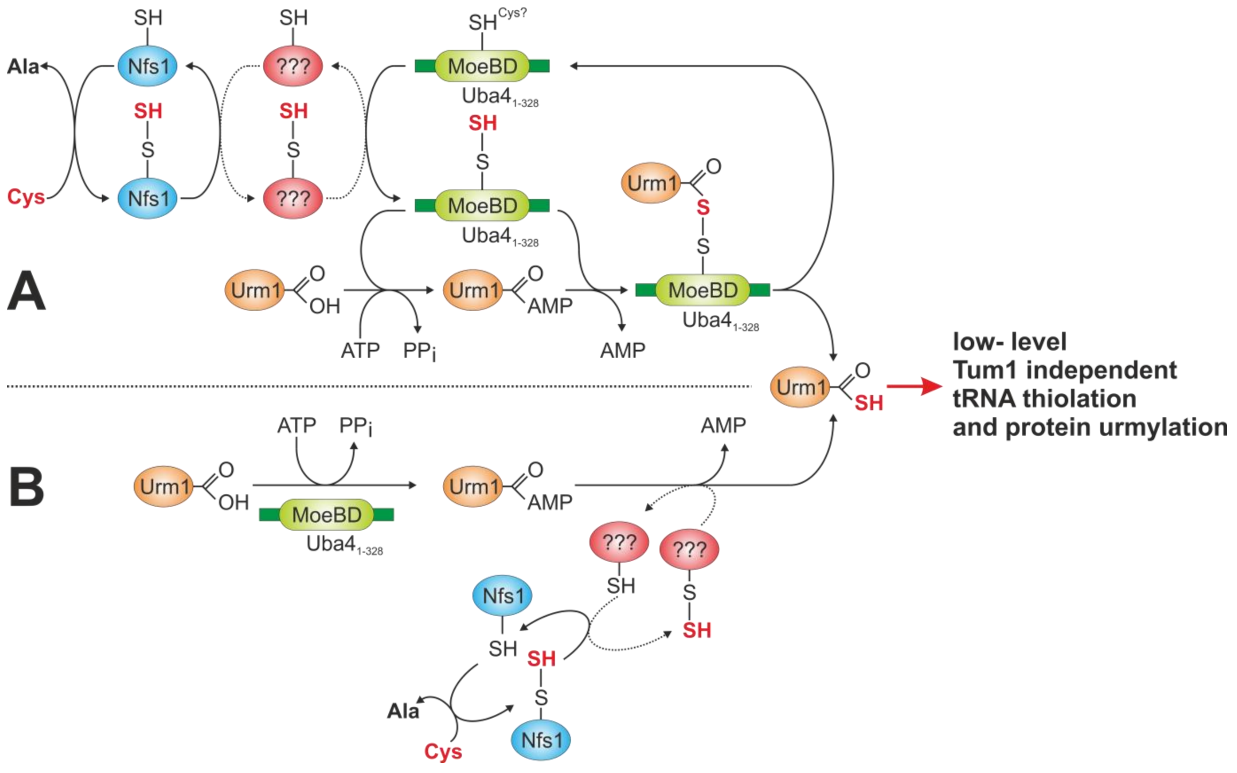

FIGURE S9: Models to explain residual contribution of Uba4$_{1\text{-}328}$ to Urm1 activation and low-level tRNA thiolation and urmylation in the absence of both Tum1 and the RHD. (A) Uba4$_{1\text{-}328}$ is still directly involved in the sulfur transfer to Urm1. The MoeBD region of the truncated activator Uba4$_{1\text{-}328}$ adenylates Urm1. Adenylated Urm1 forms an acyl-disulfide bond with a persulfide, which was previously formed at a cysteine residue within the MoeBD of Uba4$_{1\text{-}328}$. Persulfide formation could involve Nfs1 and/or an unknown (?) Tum1-independent S-transferase. **(B)** Sulfur incorporation into Urm1 is not directly mediated by Uba4$_{1\text{-}328}$. In this case Uba4$_{1\text{-}328}$ is only involved in E1-like adenylation of Urm1. The thiocarboxylation of Urm1 could be directly mediated by Nfs1 or an S-transferase alternative to Tum1 (?). Both routes (A,B) may generate Urm1-COSH levels sufficient for the observed low residual tRNA thiolation and urmylation capacities (see Fig. 5 and Fig. S7) of cells producing the MoeBD region (without the RHD) on Uba4$_{1\text{-}328}$.

4. Supplementary References

1. Klassen R, Grunewald P, Thüring KL, Eichler C, Helm M, and Schaffrath R (**2015**). Loss of anticodon wobble uridine modifications affects $tRNA^{Lys}$ function and protein levels in *Saccharomyces cerevisiae.* **PLoS ONE** 10(3): e0119261.

2. Jüdes A, Ebert F, Bär C, Thüring KL, Harrer A, Klassen R, Helm M, Stark MJ, and Schaffrath R (**2015**). Urmylation and tRNA thiolation functions of ubiquitin-like Uba4·Urm1 systems are conserved from yeast to man. **FEBS Lett** 589(8): 904-909.

3. Christianson TW, Sikorski RS, Dante M, Shero JH, and Hieter P (**1992**). Multifunctional yeast high-copy-number shuttle vectors. **Gene** 110(1): 119-122.

4. Gietz RD and Sugino A (**1988**). New yeast-*Escherichia coli* shuttle vectors constructed with *in vitro* mutagenized yeast genes lacking six-base pair restriction sites. **Gene** 74(2): 527-534.

5. Furukawa K, Mizushima N, Noda T, and Ohsumi Y (**2000**). A protein conjugation system in yeast with homology to biosynthetic enzyme reaction of prokaryotes. **J Biol Chem** 275(11): 7462-7465.
